# Pseudocellulitis Due to Antineoplastic Therapy: A Case of Atypical Presentation and Recent Developments

**DOI:** 10.7759/cureus.84688

**Published:** 2025-05-23

**Authors:** Shiva Salmasi, Zahra Gafarzadeh, Ruba Alchaikh Hassan, Constantin A Dasanu

**Affiliations:** 1 Internal Medicine, Eisenhower Medical Center, Rancho Mirage, USA; 2 Oncology and Hematology, Lucy Curci Cancer Center, Eisenhower Medical Center, Rancho Mirage, USA

**Keywords:** antineoplastic agents, gemcitabine, gemcitabine-induced pseudocellulitis, oncology, pharmacology and therapeutics, pseudocellulitis

## Abstract

The term "pseudocellulitis" refers to a non-infectious, non-necrotizing inflammation of the dermis and hypodermis. A skin reaction seen in antineoplastic therapy-treated patients, it mimics cellulitis by displaying symptoms of erythema and swelling that are not caused by infection or trauma. Previously considered rare, the incidence of pseudocellulitis has been increasing due to the continuous expansion of antineoplastic armamentarium. The most common drugs associated with pseudocellulitis are gemcitabine and pemetrexed, but some of the newer agents have also been reported as potential culprits. Patients of Caucasian extraction tend to be affected more often than other ethnic groups, with a predilection for men. Herein, we present an atypical case of pseudocellulitis due to gemcitabine, review the current literature, and shed more light on this still incompletely understood clinical entity. We also expand the knowledge on etiopathogenesis, clinical presentation, diagnosis, misdiagnosis, and management. The latter typically involves conservative measures such as limb elevation and cooling, along with the patient's reassurance. Correct and timely diagnosis is essential to avoid unnecessary antibiotic use, reducing the risk of antimicrobial resistance, hospitalizations, and societal costs.

## Introduction

Chemotherapeutic agents have been associated with a variety of cutaneous reactions, including radiation recall dermatitis, hypersensitivity reactions, and erysipeloid reactions [1]. The term "pseudocellulitis" is used to describe an uncomplicated, non-necrotizing inflammation of the dermis and hypodermis due to a noninfectious etiology. Cases of pseudocellulitis have been shown to occur with increasing frequency in recent years [1-4].

## Case presentation

A 63-year-old Caucasian woman of Ashkenazi Jewish descent presented with altered mental status. Past medical history included primary biliary cirrhosis, diverticulosis, osteoporosis, polymyalgia rheumatica, chronic constipation, gastroesophageal reflux disease, chronic lower back pain, and major depressive disorder. Family history was remarkable for esophageal cancer in her brother at the age of 29 and lung and prostate cancer in her father at the age of 88. Both her paternal grandparents had cancer: her grandmother with pancreatic cancer at age 80 and her grandfather with breast cancer at the age of 74. Social history was negative for tobacco smoking or alcohol excess.

**Figure 1:**
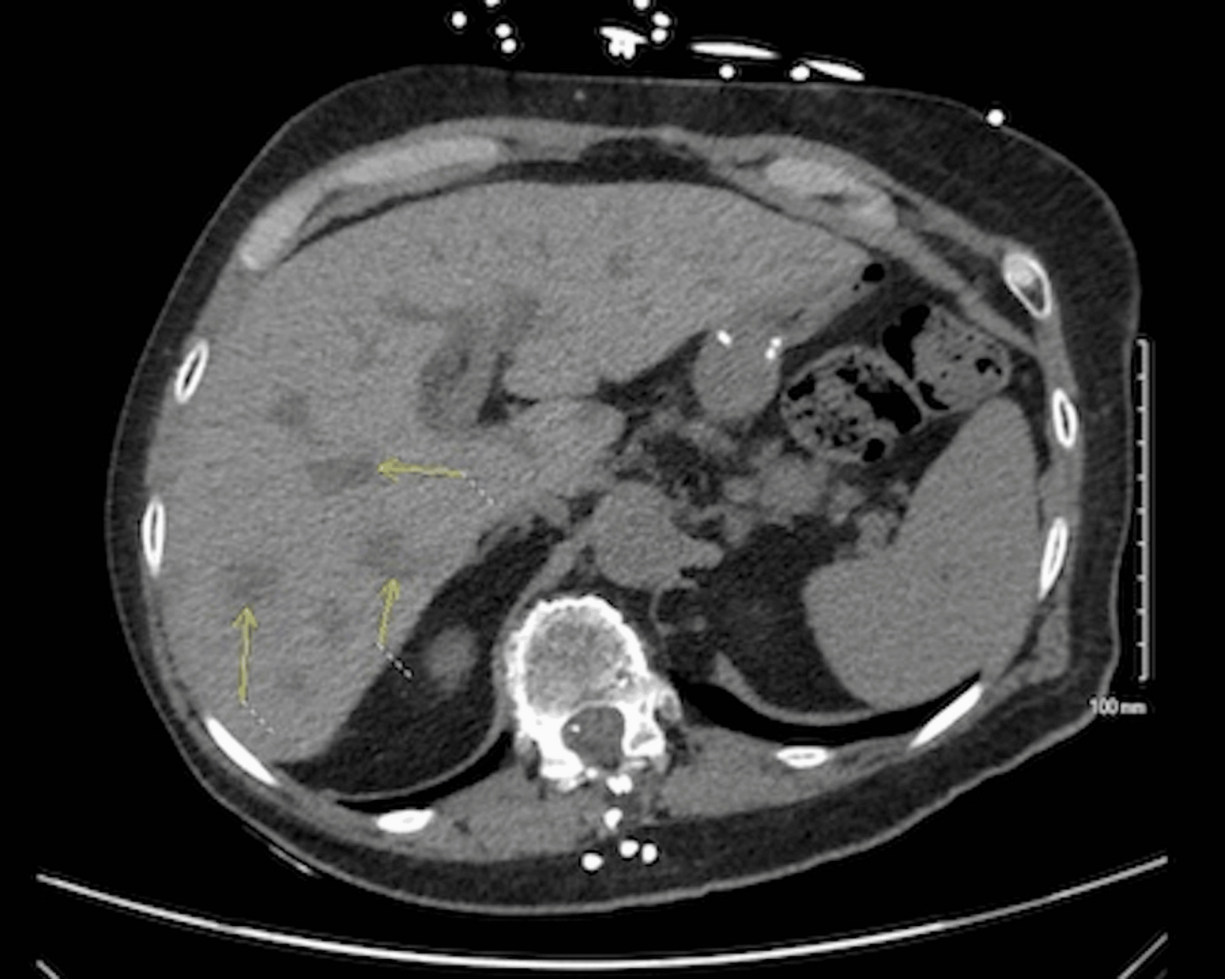
CT scan of the liver, showing multiple liver masses.

On physical exam, she was moderately pale and had mild hepatomegaly. Blood counts and a comprehensive metabolic panel were normal. Serum carbohydrate antigen 19-9 (CA 19-9) measured at 24 U/mL (reference range: 0.0-35.0 U/mL), carcinoembryonic antigen (CEA) at 0.4 ng/mL (reference range: ≤3.0 ng/mL), and alpha-fetoprotein (AFP) at 3.2 ng/mL (reference range: 0.0-6.4 ng/mL) were normal, but CA-125 was elevated at 105 U/mL (reference range: 0.0-35.0 U/mL). Further evaluation with a contrast CT scan of the abdomen showed multiple liver masses (Figure 1). A liver biopsy showed metastatic, poorly differentiated carcinoma with possible primary sites of upper gastrointestinal, pancreatobiliary, or bladder cancer. Bioteranostics cancer type ID showed a 90% probability for pancreaticobiliary adenocarcinoma. FoundationOne liquid panel (Foundation Medicine, Boston, MA, USA) showed microsatellite-stable malignancy. She was started on cisplatin 50 mg/m^2^ on day one, along with gemcitabine 1000 mg/m^2^ on days one and eight, every 21 days, and achieved a good partial response after four cycles.

**Figure 2:**
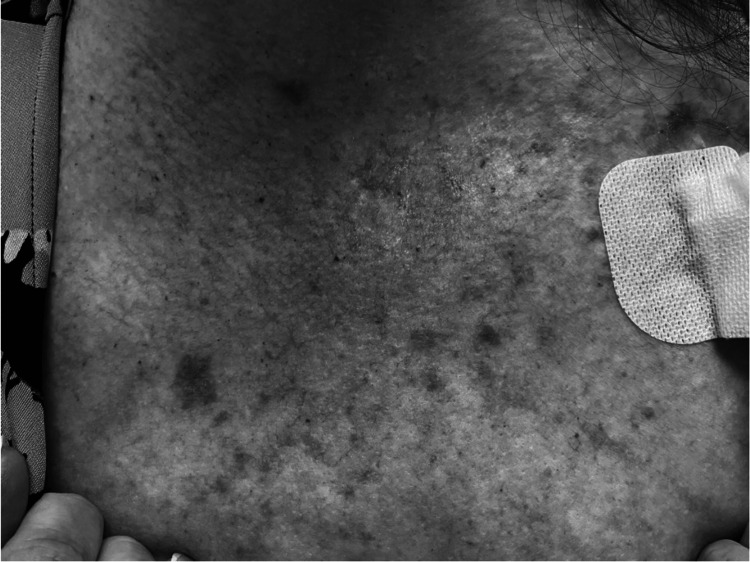
Pseudocellulitis affecting the skin on the upper chest in the index patient.

One week after the first therapy cycle, she developed a patchy erythematous rash over her chest and upper aspect of her breasts. The patient reported no fever, chills, or other systemic symptoms. On examination, she had confluent, macular, erythematous skin lesions on her chest, with the largest measuring 1.0 cm in diameter (Figure 2). The lesions blanched with applied pressure. Pseudocellulitis related to gemcitabine was promptly suspected, and no additional treatment was provided for this condition. Her symptoms improved without any intervention. Systemic chemotherapy was continued. Causality assessment between gemcitabine use and pseudocellulitis via the Naranjo nomogram questionnaire yielded a score of 5 (Table 1).

**Table 1:** The Naranjo adverse drug reaction (ADR) probability scale questionnaire. The Naranjo criteria classify the probability that an adverse event is related to drug therapy based on a list of weighted questions, which examine factors such as the temporal association of drug administration and event occurrence, alternative causes for the event, drug levels, dose-response relationships, and previous patient experience with the medication. The ADR is assigned to a probability category from the total score as follows: definite if the overall score is 9 or greater, probable for a score of 5-8, possible for 1-4, and doubtful if the score is 0. The Naranjo criteria do not take into account drug-drug interactions. Drugs are evaluated individually for causality, and points are deducted if another factor may have resulted in the adverse event, thereby weakening the causal association [5].

To assess the adverse drug reaction, please answer the following questionnaire and give the pertinent score	Yes	No	Do not know	Score
Are there previous conclusive reports on this reaction?	+ 1	0	0	+1
Did the adverse event occur after the suspected drug was administered?	+2	-1	0	+2
Did the adverse reaction improve when the drug was discontinued or a specific antagonist was administered?	+1	0	0	0
Did the adverse reaction reappear when the drug was readministered?	+2	-1	0	-1
Are there alternative causes (other than the drug) that could have on their own caused the reaction?	-1	+2	0	+2
Did the reaction reappear when a placebo was given?	-1	+1	0	0
Was the drug detected in the blood (or other fluids) in concentrations known to be toxic?	+1	0	0	0
Was the reaction more severe when the dose was increased or less severe when the dose was decreased?	+1	0	0	0
Did the patient have a similar reaction to the same or similar drugs in any previous exposure?	+1	0	0	0
Was the adverse event confirmed by any objective evidence?	+1	0	0	+1
Total				5

## Discussion

Pseudocellulitis: risk factors and etiopathogenesis

A skin reaction encountered in chemotherapy-exposed patients, pseudocellulitis mimics cellulitis by displaying symptoms of erythema and swelling but is not caused by infection or trauma. Its incidence has been increasing due to the continuous approval of new agents against cancer. By far, the most common traditional chemotherapy agent linked with skin reaction has been gemcitabine.

Gemcitabine (2,2-difluorodeoxycytidine) is currently being used for the treatment of many solid malignancies, including non-small-cell lung cancer, pancreatic cancer, biliary cancers, breast cancer, ovarian cancer, and malignant mesothelioma [1-4]. Gemcitabine is activated within cells into difluorodeoxycytidine triphosphate, which disrupts DNA synthesis by terminating chain elongation and inhibiting DNA polymerase and ribonucleotide reductase. The precise cause of gemcitabine-induced pseudocellulitis is not fully understood, but one theory suggests that impaired lymphatic drainage allows the drug to diffuse into interstitial fluid. This process may lead to the relatively lipophilic gemcitabine accumulating in subcutaneous tissues, which could alter capillary permeability and trigger a localized response. Although the exact mechanism is unclear, venous stasis may also play a role in pseudocellulitis, shedding light on the potential pathophysiology behind this reaction [4]. According to some researchers, direct vascular toxicity to the skin capillaries is suspected to be the culprit [6]. To complicate things further, gemcitabine was shown to worsen peripheral vascular disease in some patients and, in this way, potentially exacerbate the skin reactions. Dasanu and Bockorny [6] suggested that lower extremity pseudocellulitis caused by gemcitabine may be a consequence of both direct vascular toxicity to skin vasculature and a temporary exacerbation of peripheral vascular disease in affected patients.

**Figure 3:**
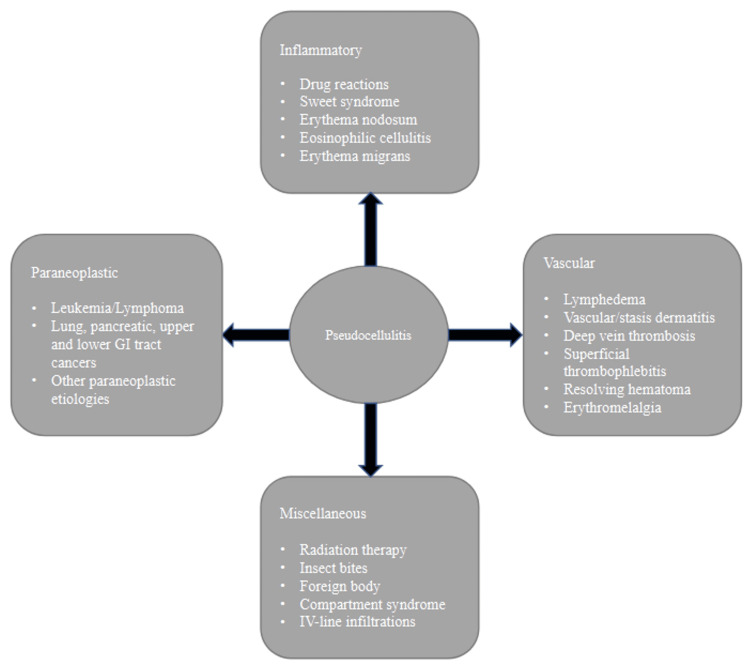
Pseudocellulitis: pharmacologic and non-pharmacologic etiologies. Image created by the authors.

Pseudocellulitis has also been described with the use of pemetrexed, which is the second most common agent associated with this skin condition [2,7,8]. Other antineoplastic agents linked with pseudocellulitis in recent years are listed in Table 2. Non-pharmacologic etiologies of pseudocellulitis include inflammatory (e.g., Sweet syndrome, erythema nodosum), vascular (e.g., lymphedema, thrombosis), paraneoplastic (e.g., leukemia, lymphoma), and miscellaneous (e.g., radiation therapy, IV line infiltrations), which are listed in Figure 3.

**Table 2:** Pharmacologic agents linked with pseudocellulitis. Source: [9]

Class of anticancer drugs	Agent	Prevalence/level of evidence
Traditional cytotoxic chemotherapy	Gemcitabine	Most common (several case series and multiple case reports)
Traditional cytotoxic chemotherapy	Pemetrexed	Second most common (a case series and isolated reports)
Traditional cytotoxic chemotherapy	Carboplatin	Case report
Traditional cytotoxic chemotherapy	Paclitaxel	Case report
Targeted agents	Lorlatinib, rituximab	Case report
Proteasome inhibitors	Bortezomib	Case report
Antibody-drug conjugates	Enfortumab vedotin	Case report
Immune checkpoint inhibitors	Atezolizumab	Case report

Skin toxicity of gemcitabine

The common side effects of gemcitabine include myelosuppression, gastrointestinal disturbances, and liver enzyme elevation [10,11]. Cutaneous reactions to gemcitabine are frequent, occurring in up to 25% of treated patients. Gemcitabine has been associated with erythematous or papulloerythematous rash, alopecia, pruritus, hypersensitivity reactions, skin hyperpigmentation, and skin necrosis. There are isolated reports of livedo reticularis, scleroderma-like changes, Sweet syndrome, and toxic epidermal necrolysis [12-14]. The most common cutaneous toxicities are mild papulloerythematous rash and pruritus [1,15,16]. Pseudocellulitis in the absence of prior radiation exposure is a relatively rare side effect of gemcitabine [4].

Clinical features of pseudocellulitis

Similar to cellulitis, patients usually show localized inflammation of the dermis and hypodermis, with symptoms often appearing within two days of starting gemcitabine treatment. Pseudocellulitis most commonly involves the lower extremities [3]. Bilateral (symmetrical) reactions are more common than unilateral [4]. However, atypical presentations also occur, as seen in our patient who had an atypical presentation involving symmetrical skin on the chest. Patients generally present without fever or systemic symptoms of infection. Lesions usually fade without specific therapy within 5-14 days, and reoccurrence is rather infrequent [6,17].

Management

While pseudocellulitis generally follows a self-limiting course, it may sometimes necessitate discontinuing gemcitabine and providing symptomatic treatment, such as non-steroidal anti-inflammatory drugs or topical steroids [18]. Discontinuation of gemcitabine eventually results in the resolution of the edema, softening of the skin, and partial reversibility of the fibrotic process [19,20]. Gemcitabine can be safely continued in most patients with this complication, though recurrence of the rash can be seldomly seen following repeated doses [15]. The cisplatin-gemcitabine regimen was continued as our patient had responded well to this therapy. The patient's lesions improved within 10 days and did not reoccur with subsequent cycles.

Differential diagnosis

Over 10% of patients diagnosed with cellulitis actually suffer from other conditions. Classic signs of cellulitis include redness, localized warmth, tenderness, and swelling of the skin. A recent injury or pain in the affected area, along with signs of leukocytosis, may also support a cellulitis diagnosis. Typically, cellulitis appears on one side with smooth, indistinct borders; in contrast, a symmetric or diffusely spread pattern is likely to suggest another condition. Additional indicators of cellulitis are underlying immunosuppression, rapid symptom progression, previous similar infections, systemic signs (such as fever or elevated white blood cell count), recent travel or outdoor activities, and health issues like diabetes or peripheral vascular disease. Alternatively, a slow-developing, long-lasting course or a history of ineffective antibiotic treatment points to a condition other than cellulitis [21-24].

In patients receiving chemotherapy who present with localized erythema and swelling of the lower legs, potential diagnoses include infectious cellulitis, erysipelas, eosinophilic cellulitis, acute lymphedema, superficial venous thrombosis, allergic contact dermatitis, lipodermatosclerosis, stasis dermatitis, erythema nodosum, cutaneous gout, and bursitis. Bilateral involvement or a poor response to antibiotics when cellulitis is suspected often points to a non-infectious cause. Eosinophilic cellulitis may appear suddenly as red plaques, typically without fever, similar to pseudocellulitis; however, eosinophilic cellulitis often presents with intense itching, and plaques may resolve with a greenish tint, which can aid diagnosis. Up to half of these patients show peripheral eosinophilia [7,21,25,26].

Patients with pseudocellulitis usually exhibit lower extremity edema and a slight induration of the involved sites. Unlike cellulitis, fever and leukocytosis are usually absent; however, gemcitabine can cause drug-induced fever and myelosuppression, which may complicate the diagnosis [3,17].

Misdiagnosis and consequences

There is limited data on the cost and complications from misdiagnosed cellulitis. In a study by Weng et al. [28], involving 259 patients, 30.5% were misdiagnosed with cellulitis, and more than half of these misdiagnosed patients were admitted to the inpatient wards primarily for treatment of cellulitis. Of the 52 patients, 44 (84.6%) did not require hospitalization based on their final diagnosis, and 48 (92.3%) received antibiotics that were ultimately unnecessary [28]. Misdiagnosing these cases can expose patients to needless antibiotic treatments, hospital stays, and disruptions to chemotherapy, all of which contribute to significantly elevated healthcare costs [3,28,29].

Studies indicate that improper treatment and hospitalization for patients with misdiagnosed lower extremity cellulitis may lead to over 9,000 hospital-acquired infections, including approximately 1,000 to 5,000 symptomatic cases of *Clostridium difficile* infection [30]. In addition, unnecessary antibiotic exposure increases the risk for antibiotic resistance; therefore, awareness of this reaction is critical in order to avoid unnecessary antibiotics and costly diagnostic workups [31]. A retrospective chart review between 2016 and 2022 showed that 17/31 patients (56%) experienced pseudocellulitis due to gemcitabine, while 12.9% of cases were attributed to pemetrexed use. Most of the affected patients were Caucasian men. A total of 24 patients had received systemic antibiotics before being referred to dermatology [9].

## Conclusions

It is extremely important to consider pseudocellulitis in the differential diagnosis when evaluating patients with skin rash, particularly in those undergoing systemic chemotherapy. These skin reactions can present with erythema and swelling, mimicking infectious cellulitis. Misdiagnosis can lead to inappropriate antibiotic therapy and hospitalizations, which contributes to the risk of antimicrobial resistance and exposes patients to potential adverse effects. In contrast, the management of pseudocellulitis typically involves observation and conservative measures, such as limb elevation and cooling, without the need for antibiotics or aggressive interventions. Correct identification and management of pseudocellulitis can thus reduce healthcare costs and improve patient outcomes.
